# Determinants of early initiation of breastfeeding in Peru: analysis of the 2018 Demographic and Family Health Survey

**DOI:** 10.4178/epih.e2019051

**Published:** 2019-12-25

**Authors:** Akram Hernández-Vásquez, Horacio Chacón-Torrico

**Affiliations:** 1 Universidad San Ignacio de Loyola, Vicerrectorado de Investigación, Centro de Excelencia en Investigaciones Económicas y Sociales en Salud, Lima, Peru; 2 Facultad de Medicina, Universidad Científica del Sur, Lima, Peru

**Keywords:** Breastfeeding, Newborn, Health surveys, Peru

## Abstract

**OBJECTIVES:**

Early initiation of breastfeeding (EIBF) is one of the most cost-effective strategies to reduce neonatal mortality. We sought to determine the prevalence and determinants of EIBF in Peru.

**METHODS:**

We performed a cross-sectional analytical study of the 2018 Peruvian Demographic and Family Health Survey as a secondary data source. In total, 19,595 children born during the 5 years prior to the survey were included in the study. The dependent variable (EIBF status), socio-demographic variables, and pregnancy-related variables were analyzed using a multivariate logistic regression model to identify the determinants of EIBF.

**RESULTS:**

The prevalence of EIBF in the study population was 49.7%. Cesarean deliveries were associated with a lower likelihood of EIBF (adjusted odds ratio [aOR], 0.06; 95% confidence interval [CI], 0.05 to 0.07) than were vaginal deliveries. Newborns born at public health centers (aOR, 1.37; 95% CI, 1.15 to 1.65) had a higher rate of EIBF than those not born at public or private health centers. Women from the jungle region (aOR, 2.51; 95% CI, 2.17 to 2.89) had higher odds of providing EIBF than those from the coast. Mothers with more than a secondary education (aOR, 0.65; 95% CI, 0.55 to 0.76) were less likely to breastfeed during the first hour of the newborn’s life than women with primary or no education.

**CONCLUSIONS:**

More than half of Peruvian children do not breastfeed during the first hour after birth. The major determinants of EIBF status were the delivery mode and the region of maternal residence. Strategies are needed to promote early breastfeeding practices.

## INTRODUCTION

Although neonatal mortality (NM) worldwide decreased by half between 1990 and 2017, it is projected that by 2030, 27.8 million newborns will die during the first month of life [1]. In order to reduce neonatal deaths, the World Health Organization (WHO) recommends a series of essential, cost-effective, and easy-to-implement practices [2]. These practices include hygienic handling of the umbilical cord, thermal control, and early lactation. Evidence based on high-quality data has confirmed the benefits of early lactation for NM [3], and it is estimated that the implementation of large-scale breastfeeding promotion programs could prevent over 11.6% of newborn deaths and cause a reduction of over 21.9 million disability-adjusted life years [4].

Early initiation of breastfeeding (EIBF) is defined as the intake of breast milk by the newborn within the first hour after birth [5]. The main benefits of EIBF include stimulating colostrum production, reducing postpartum hemorrhage, and promoting exclusive long-term breastfeeding [6,7]. However, it should be noted that despite the position of the WHO on the benefits of EIBF, several studies have failed to show a positive relationship between EIBF and exclusive breastfeeding [8,9]. It has also been estimated that up to 22% of neonatal deaths can be prevented with EIBF [10]. Nonetheless, it has been reported that only around half of newborns worldwide are breastfed within the first hour of life [11]. Likewise, a study of 57 low-income and middle-income countries showed that only 39% of newborns engaged in EIBF [12].

Several studies have reported that some socio-demographic characteristics, such as place of residence, maternal education level, socioeconomic status, and place of birth, are associated with EIBF [13,14]. Cultural beliefs and traditional feeding practices have also been described as important barriers to EIBF [15]. However, making generalizations regarding the determinants of EIBF is difficult due to the presence of considerable regional differences [14]. For countries in Latin America and the Caribbean, there is still little evidence on this issue. Studies conducted in these regions have reported that the prevalence of EIBF among newborns delivered in private-sector facilities was 45.2%, as opposed to 62.8% among those born at public-sector institutions [12].

In Peru, the 2018 Demographic and Family Health Survey (ENDES, for its acronym in Spanish) reported that over 49.7% of the children born during the 5 years prior to the survey were breastfed during the first hour after birth. In addition, a multicenter study reported that the prevalence of EIBF in hospitals in Peru was the lowest (17.7%) of 24 low-income and middle-income countries in Africa, Asia, and Latin America [16]. Peru is a middle- to high-income country, and despite having reached the goal of reducing NM as required by the Millennium Goals for 2015, it still exhibits many inequalities that underlie access to most indicators of child and neonatal health [17]. These differences can be attributed to the geographic and socioeconomic diversity of Peru.

Given the suboptimal rate of EIBF in Peru and the scarce evidence regarding its determinants, this problem must be assessed. For this reason, the aim of the present study was to analyze the socio-demographic and maternal factors related to EIBF in newborns in Peru during the 5 years prior to the 2018 ENDES.

## MATERIALS AND METHODS

### Design and population study

We conducted a secondary analysis of data from the 2018 ENDES. The ENDES was carried out by the *Instituto Nacional de Estadística e Informática* (INEI) of Peru. ENDES data are freely available and can be obtained from the INEI web portal (http://iinei.inei.gob.pe/microdatos/). The ENDES used a multi-stage stratified random sampling and includes individuals living in the selected households. The details of the methodology of the survey can be consulted in the final technical report [18].

Members of a total of 21,960 households from urban and rural places of the 24 departments of Peru and the province of Callao were interviewed. For the 2018 ENDES, 34,971 women between 15 and 49 years of age and 23,983 children under 5 years old residing in the selected households were identified.

### Variables and measurements

According to the WHO, EBIF is defined as breastfeeding within the first hour after birth [5]. In the 2018 ENDES, women were asked about the initiation of breastfeeding for children born in the 5 years prior to the survey. Responses of fewer than 24 hours were recorded in hours. A binary variable was then created and categorized as: (1) EIBF if a child was breastfed immediately or within 1 hour after birth, or (2) non-EIBF if a child was breastfed after 1 hour.

The following independent variables were included in the analysis: age of the woman in years (15-24, 25-34, or 35-49), marital status (never married, separated/divorced/widowed, or married/cohabiting), level of education (no formal school/primary, secondary, or higher), wealth index quintiles (poorest, poorer, middle, richer, or richest), region of residence (Peru is divided into 3 regions: the coastal region, near the Pacific coastal line [including Lima, the country’s capital city]; the highlands region of the Andes; and the jungle, where the Amazon rainforest is located), place of residence (urban or rural), cesarean section (yes or no), place of delivery (private health facility, public health facility, or other), birth order (first, second or third, or fourth or higher), size of the child at birth (small, medium, or large), sex of the newborn (female or male), type of pregnancy (multiple or single), number of prenatal visits (0-3, 4-7, or 8 or more), utilization of breastfeeding training (yes or no), ethnic self-identification (White/mixed-race/other, Native, or Black/Brown/Zambo), and head of the household (yes or no). The selection and inclusion of these variables was based on an epidemiological criterion and on variables reported in previous ENDES-based studies [19-23].

### Statistical analysis

All analyses were conducted using Stata version 14.2 (Stata Corp., College Station, TX, USA), and the svy command was used to adjust for sampling weights and clustering. Demographic and socioeconomic characteristics, as well as the outcome variable, were described by absolute frequencies and weighted proportions with 95% confidence intervals [Cis]. The chi-square test was utilized to assess the associations between the explanatory variables and the outcome variable.

Crude odds ratios (ORs) and adjusted odds ratios (aORs) with 95% CIs were also calculated. A logistic regression model was used to measure the association between the study factors and EIBF status. A bivariate logistic regression (crude analysis) was carried out among the variables of interest. Independent variables with p-values <0.20 in the bivariate analysis and factors known from the literature to predict the outcome variable were included in the multiple logistic regression. The OR values with 95% CIs for both models are presented. Multicollinearity was assessed using the variance inflation factor. A p-value <0.05 was considered to indicate statistical significance, and p-values were not corrected for multiple testing.

### Ethics statement

This study did not require the approval of an ethics committee because it was an analysis of a de-identified secondary dataset of the 2018 ENDES, which is freely and publicly available. The aim of these annually executed surveys under the Demographic and Health Surveys model [24] is to obtain data on a wide range of nationwide development indicators by a governmental agency.

## RESULTS

From the 2018 ENDES population total of 19,696 children, our analysis included 19,595 children born during the 5 years prior to the survey and for whom complete information was available. Table 1 shows the distribution of the study population according to the variables studied. Approximately 9 out of 10 deliveries (92.7%) occurred in a private or public health care facility. The mean maternal age at the time of the survey was 30.4±7.1 years. The most common marital status was married/cohabiting (84.5%), and the most common places and regions of residence were urban places and the coast region, respectively, with 75.0% of participants residing in an urban place and 56.0% from the coastal region.

Approximately half of the women (49.7%) surveyed reported having breastfed their newborn during the first hour after birth. This proportion varied significantly based on the characteristics evaluated, with the exceptions of marital status and whether the woman was the head of the household (Table 2). With regard to wealth index, immediate breastfeeding ranged from 70.6% in the lowest wealth quintile to 27.5% in the highest quintile. Likewise, there was a major difference in the use of EIBF according to delivery mode, with a higher prevalence of EIBF following vaginal delivery (71.1%) than cesarean section. EIBF also varied greatly by education level, with EIBF being engaged in by 68.7% of women with primary or no education and by 36.3% of women with more than a secondary education.

Table 3 shows the raw and adjusted binary logistic regression models between the independent variables and EIBF status. Women who experienced a cesarean delivery were 94% less likely to breastfeed during the first hour after delivery than women who experienced a vaginal birth, thereby showing delivery mode to be the strongest determinant in both the crude and adjusted models (OR, 0.05; aOR, 0.06). Age group was not associated with frequency of immediate breastfeeding in either the crude or aOR model. The socio-demographic variable showing the strongest association with EIBF was the region of maternal residence. As such, women from the jungle region were more likely to breastfeed during the first hour than those from the coast (aOR, 2.51; 95% CI, 2.17 to 2.89). In contrast, women who ethnically identified themselves as “Native” were 14% less likely to breastfeed immediately than women who self-identified as “White/mixed-race/other” (aOR, 0.86; 95% CI, 0.76 to 0.97).

With regard to the pregnancy and childbirth variables, women who experienced a single birth were more likely (aOR, 2.81; 95% CI, 1.35 to 5.85) to engage in EIBF than those who experienced multiple births. In addition, the size of the newborn was also moderately associated with a higher probability of EIBF in the adjusted model, with aOR values of 1.37 (95% CI, 1.21 to 1.54) and 1.20 (95% CI, 1.04 to 1.39) for medium and large infants, respectively, compared to small infants. Finally, birth order which is related to the woman’s parity, was also associated with a higher probability of EIBF, with women with 4 or more deliveries being 21% more likely to perform EIBF than those delivering their first child (aOR, 1.21; 95% CI, 1.01 to 1.45).

## DISCUSSION

The main objective of this study was to identify the determinants of EIBF among 15-year-old to 49-year-old women in Peru using the 2018 ENDES as the data source. According to this dataset, approximately half (49.7%) of newborns were breastfed during the first hour after birth. While this constitutes a slight increase in EIBF compared to the previous year (48.2%), a constant decline has been observed since 2013, when the frequency of EIBF peaked at 55.6% [18]. The results of the present study show that the type of pregnancy and maternal region of residence had the greatest association with EIBF.

Multiple studies have reported the mode of delivery to be one of the major determinants of EIBF status in newborns [20,25], with delivery by cesarean section being associated with non-compliance with immediate breastfeeding. This route of delivery induces barriers, such as a delay in skin-to-skin contact between mother and child due to anesthesia, as well as the fatigue associated with a prolonged birth [26,27]. Taking into account the progressive increase in cesarean sections both globally and locally, knowledge of the negative effects of this procedure on immediate breastfeeding is important to prevent neonatal deaths.

In contrast, a lower prevalence of EIBF was observed among mothers with a higher level of education. This contrasts with the findings of studies conducted in India [19], Ethiopia [20], and Nigeria [28], which have described that the higher the education level of the mother, the higher the likelihood of EIBF. As shown in a study performed by Islam et al. [29] in Bangladesh, it is thought that other contextual factors can shape the relationship between EIBF and maternal education level. Highly educated women also have a high rate of cesarean sections, which could explain this inverse relationship in the Peruvian population. Despite these results, we believe that education is an important factor associated with higher EIBF rates and that it should be taken into consideration in public health care approaches aimed at promoting newborn health.

Additionally, we found that newborns of mothers from a greater wealth quintile had a lower probability of early breastfeeding. This contrasts with the results of several studies in which a higher socioeconomic level was a positive determinant of EIBF [20,26]. It is possible that the association found in this study was determined by practices conducted by the Peruvian population. Another possible explanation is that greater purchasing power allows wealthier mothers to acquire pre-milk supplementation for newborns.

In contrast with the results of previous studies [19], urban or rural place of residence was not a determinant of EIBF status. However, it was observed that newborns of women originating from the jungle region were much more likely to receive immediate breastfeeding than those of women from the coast. It is thought that other cultural and social determinants related to geographical location influence EIBF. These determinants may include low exposure to pro-formula feeding propaganda and the low socioeconomic status associated with the jungle region of residence [30,31].

Several studies have described how access to health services, mainly prenatal care, is a determinant of EIBF [19,32]. However, in our study, this association was not significant. Possible explanations may be poor breastfeeding counseling during antenatal visits that fail to promote this practice, the fragmentation of the Peruvian health system, or the lack of national strategies for the promotion of immediate breastfeeding. It has been reported that the promotion of breastfeeding in health centers in Peru is influenced by the over-demand for health services, poor staff training, and the influence of the formula industry [33]. It is imperative to structure health programs that provide information to pregnant women about the benefits of EIBF. In light of the information collected, the efforts made at promoting EIBF at the primary care level are not effective enough.

Birth order of 3 or more previous deliveries was moderately associated with a higher prevalence of EIBF. Similar results were also found in Malawi; in that study, birth order of at least 1 previous delivery was associated with an aOR of 1.30 (95% CI, 1.06 to 1.67) compared to nulliparous women [34]. It is known that nulliparous women generally have little to no knowledge of pregnancy and childbirth, which could be an important factor influencing attitudes and practices during the first hour after birth [19]. According to the results obtained in the present study, nulliparous women should be prioritized as recipients of breastfeeding counseling and immediate newborn care practices.

Similarly, deliveries at health centers were associated with a higher prevalence of EIBF, especially those at private centers. While previous studies have described lower rates of EIBF associated with deliveries at health centers in Bangladesh [28], a positive association with EIBF has alternatively been reported [34]. Indeed, childbirth care by qualified personnel is designed to encourage pregnant women to start breastfeeding. We found a stronger association with EIBF at private health centers, which likely provide higher-quality training and reinforcement of breastfeeding counseling.

The design of this study was cross-sectional, which was one of the major limitations given its incapacity for establishing causal associations. In addition, when using a secondary data sources, not all potential confounding factors, such as cultural practices or access to lacteal supplementation products, were monitored. An additional bias is that of memory, since the information collected was based entirely on the ability of the women to remember the time at which breastfeeding was initiated. Despite these limitations, the ENDES uses standardized procedures and is performed by trained examiners, which, along with its complex sample design, guarantee the quality, adequate measurement, and national representativeness of the information.

This study evaluated the demographic and health factors associated with EIBF in a representative sample of Peruvian women aged 15 to 49 years. Factors such as mode of delivery, education level, region of residence, place of delivery, birth order, type of pregnancy, and ethnic self-identification were associated with EIBF status. Knowledge of these specific factors in both Peru and countries with similar characteristics in Latin America and the Caribbean may be useful for the design, planning, and execution of breastfeeding promotion strategies aimed at directly and permanently affecting neonatal health.

## Figures and Tables

**Table 1.** Characteristics of the women and children included in the study (n=19,595)

Characteristics	n	Weighted %^1^
Early initiation of breastfeeding		
No	9,088	50.3
Yes	10,507	49.7
Age (yr)		
15-24	4,710	22.9
25-34	9,037	46.5
35-49	5,848	30.6
Marital status		
Never married	1,028	5.1
Separated/divorced/widowed	2,033	10.4
Married/cohabiting	16,534	84.5
Level of education		
No formal school/primary	4,163	19.5
Secondary	8,856	44.2
Higher	6,576	36.3
Wealth Index		
Poorest	5,519	23.6
Poorer	5,110	23.1
Middle	3,874	20.1
Richer	2,937	17.5
Richest	2,155	15.7
Region of residence		
Coast	8,373	56.0
Andean	6,598	27.4
Jungle	4,624	16.6
Place of residence		
Rural	5,830	25.0
Urban	13,765	75.0
Mode of delivery		
Vaginal delivery	13,271	64.5
Cesarean delivery	6,324	35.5
Place of delivery		
Other^2^	1,347	7.3
Private health center	1,820	14.5
Public health center	16,428	78.2
Order of birth		
1	6,156	32.9
2-3	9,824	50.3
≥4	3,615	16.8
Newborn size		
Small	4,163	20.9
Medium	10,232	51.5
Large	5,200	27.6
Newborn sex		
Female	9,577	48.5
Male	10,018	51.5
Type of pregnancy		
Multiple	186	1.1
Single	19,409	98.9
Antenatal controls		
0-3	764	3.5
4-7	4,737	23.4
≥8	14,094	73.1
Breastfeeding training		
No	5,804	30.2
Yes	13,791	69.8
Ethnic self-identification		
White/mixed-race/others	11,136	63.0
Native	6,651	26.7
Black/Brown/’’Zambo’’	1,808	10.3
Head of the household		
No	17,081	88.1
Yes	2,514	11.9

1 Estimates include the weights and Demographic and Family Health Survey sample specifications.

2 Neither private nor public health centers.

**Table 2.** Factors associated with early initiation of breastfeeding

Characteristics	Breastfeeding, %^1^		p-value^2^
	No	Yes	
Age (yr)			
15-24	43.8	56.2	<0.001
25-34	50.0	50.0	
35-49	55.3	44.7	
Marital status			0.407
Never married	50.9	49.1	
Separated/divorced/widowed	51.9	48.1	
Married/cohabiting	50.0	50.0	
Level of education			<0.001
No formal school/primary	31.3	68.7	
Secondary	47.5	52.5	
Higher	63.7	36.3	
Wealth Index			<0.001
Poorest	29.4	70.6	
Poorer	43.8	56.2	
Middle	54.5	45.5	
Richer	61.7	38.3	
Richest	72.5	27.5	
Region of residence			<0.001
Coast	60.8	39.2	
Andean	40.2	59.8	
Jungle	30.9	69.1	
Place of residence			<0.001
Rural	31.5	68.5	
Urban	56.4	43.6	
Mode of delivery			<0.001
Vaginal delivery	28.9	71.1	
Cesarean delivery	89.0	11.0	
Place of delivery			<0.001
Other^3^	27.2	72.8	
Private health center	79.1	20.9	
Public health center	47.0	53.0	
Order of birth			<0.001
1	55.7	44.3	
2-3	50.6	49.4	
≥4	38.2	61.8	
Newborn size			<0.001
Small	53.3	46.7	
Medium	47.0	53.0	
Large	53.8	46.2	
Newborn sex			0.013
Female	49.0	51.0	
Male	51.4	48.6	
Type of pregnancy			<0.001
Multiple	88.2	11.8	
Single	49.8	50.2	
Antenatal controls			<0.001
0-3	41.8	58.2	
4-7	48.1	51.9	
≥8	51.3	48.7	
Breastfeeding training			0.007
No	52.3	47.7	
Yes	49.3	50.7	
Ethnic self-identification			<0.001
White/mixed-race/others	53.6	46.4	
Native	44.1	55.9	
Black/Brown/’’Zambo’’	45.2	54.8	
Head of the household			0.865
No	50.2	49.8	
Yes	50.4	49.6	

49.6Values are presented as weighted % of the row unless otherwise indicated.

1 Estimates include the weights and Demographic and Family Health Survey sample specifications.

2 Using chi-square test statistics.

3 Neither private nor public health centers.

**Table 3.** Crude and adjusted ORs of early breastfeeding for several socioeconomic, pregnancy, and birth variables

Variables	OR (95% CI)^1^	p-value	aOR (95% CI)^1,2^	p-value
Age (yr)				
15-24	1.00 (reference)	-	1.00 (reference)	-
25-34	0.78 (0.71, 0.85)	<0.001	0.94 (0.83, 1.07)	0.378
35-49	0.63 (0.57, 0.70)	<0.001	0.89 (0.76, 1.05)	0.159
Marital status			Not included	
Never married	1.00 (reference)	-	-	-
Separated/divorced/widowed	0.96 (0.79, 1.16)	0.667	-	-
Married/cohabiting	1.04 (0.89, 1.21)	0.649	-	-
Level of education				
No formal school/primary	1.00 (reference)	-	1.00 (reference)	-
Secondary	0.50 (0.45, 0.56)	<0.001	0.72 (0.64, 0.82)	<0.001
Higher	0.26 (0.23, 0.29)	<0.001	0.65 (0.55, 0.76)	<0.001
Wealth Index				
Poorest	1.00 (reference)	-	1.00 (reference)	-
Poorer	0.53 (0.48, 0.60)	<0.001	0.94 (0.81, 1.10)	0.430
Middle	0.35 (0.31, 0.39)	<0.001	0.84 (0.70, 1.00)	0.055
Richer	0.26 (0.23, 0.29)	<0.001	0.82 (0.67, 1.01)	0.057
Richest	0.16 (0.14, 0.18)	<0.001	0.77 (0.61, 0.98)	0.033
Region of residence				
Coast	1.00 (reference)	-	1.00 (reference)	-
Andean	2.31 (2.11, 2.53)	<0.001	1.49 (1.30, 1.70)	<0.001
Jungle	3.47 (3.12, 3.86)	<0.001	2.51 (2.17, 2.89)	<0.001
Place of residence				
Rural	1.00 (reference)	-	1.00 (reference)	-
Urban	0.35 (0.32, 0.39)	<0.001	0.90 (0.78, 1.05)	0.167
Mode of delivery				
Vaginal delivery	1.00 (reference)	-	1.00 (reference)	-
Cesarean delivery	0.05 (0.04, 0.06)	<0.001	0.06 (0.05, 0.07)	<0.001
Place of delivery				
Other^3^	1.00 (reference)	-	1.00 (reference)	-
Private health center	0.10 (0.08, 0.12)	<0.001	1.18 (0.90, 1.54)	0.225
Public health center	0.42 (0.36, 0.50)	<0.001	1.37 (1.15, 1.65)	0.001
Order of birth				
1	1.00 (reference)	-	1.00 (reference)	-
2-3	1.22 (1.13, 1.33)	<0.001	1.07 (0.94, 1.21)	0.293
≥4	2.03 (1.82, 2.27)	<0.001	1.21 (1.01, 1.45)	0.040
Newborn size				
Small	1.00 (reference)	-	1.00 (reference)	-
Medium	1.29 (1.17, 1.42)	<0.001	1.37 (1.21, 1.54)	<0.001
Large	0.98 (0.88, 1.09)	0.736	1.20 (1.04, 1.39)	0.011
Newborn sex				
Female	1.00 (reference)	-	1.00 (reference)	-
Male	0.91 (0.84, 0.98)	0.013	0.93 (0.85, 1.03)	0.158
Type of pregnancy				
Multiple	1.00 (reference)	-	1.00 (reference)	-
Single	7.55 (4.43, 12.87)	<0.001	2.81 (1.35, 5.85)	0.006
Antenatal controls				
0-3	1.00 (reference)	-	1.00 (reference)	-
4-7	0.77 (0.63, 0.95)	0.013	1.03 (0.80, 1.31)	0.833
≥8	0.68 (0.56, 0.83)	<0.001	1.20 (0.94, 1.53)	0.136
Breastfeeding training				
No	1.00 (reference)	-	1.00 (reference)	-
Yes	1.13 (1.03, 1.23)	0.007	0.93 (0.83, 1.04)	0.228
Ethnic self-identification				
White/mixed-race/others	1.00 (reference)	-	1.00 (reference)	-
Native	1.47 (1.35, 1.59)	<0.001	0.86 (0.76, 0.97)	0.012
Black/Brown/’’Zambo’’	1.40 (1.23, 1.59)	<0.001	1.02 (0.87, 1.20)	0.808
Head of the household			Not included	
No	1.00 (reference)	-	-	-
Yes	0.99 (0.88, 1.11)	0.865	-	-

OR, odds ratio; CI, confidence interval; aOR, adjusted odds ratio.

1 Estimates include the weights and Demographic and Family Health Survey sample specifications.

2 Adjusted by all the variables shown in the column that obtained a p-value less than 0.2 in the crude analysis.

3 Neither private nor public health centers.
